# The effect of high prevalence of missing data on estimation of the coefficients of a logistic regression model when using multiple imputation

**DOI:** 10.1186/s12874-022-01671-0

**Published:** 2022-07-18

**Authors:** Peter C. Austin, Stef van Buuren

**Affiliations:** 1 ICES, G106, 2075 Bayview Avenue, Toronto, M4N 3M5 ON Canada; 2 Institute of Health Policy, Management, and Evaluation, University of Toronto, Toronto, ON Canada; 3 Sunnybrook Research Institute, Toronto, ON Canada; 4 University of Utrecht, Padualaan 14, 3584 CH Utrecht, The Netherlands; 5 Netherlands Organisation for Applied Scientific Research TNO, Leiden, The Netherlands

**Keywords:** Missing data, Multiple imputation, Monte Carlo simulations

## Abstract

**Background:**

Multiple imputation is frequently used to address missing data when conducting statistical analyses. There is a paucity of research into the performance of multiple imputation when the prevalence of missing data is very high. Our objective was to assess the performance of multiple imputation when estimating a logistic regression model when the prevalence of missing data for predictor variables is very high.

**Methods:**

Monte Carlo simulations were used to examine the performance of multiple imputation when estimating a multivariable logistic regression model. We varied the size of the analysis samples (*N* = 500, 1,000, 5,000, 10,000, and 25,000) and the prevalence of missing data (5–95% in increments of 5%).

**Results:**

In general, multiple imputation performed well across the range of scenarios. The exceptions were in scenarios when the sample size was 500 or 1,000 and the prevalence of missing data was at least 90%. In these scenarios, the estimated standard errors of the log-odds ratios were very large and did not accurately estimate the standard deviation of the sampling distribution of the log-odds ratio. Furthermore, in these settings, estimated confidence intervals tended to be conservative. In all other settings (i.e., sample sizes > 1,000 or when the prevalence of missing data was less than 90%), then multiple imputation allowed for accurate estimation of a logistic regression model.

**Conclusions:**

Multiple imputation can be used in many scenarios with a very high prevalence of missing data.

## Background

Missing data is common in biomedical and epidemiological research. Missing data occurs when the value of a variable is recorded for some subjects in the sample, but not for all subjects. Multiple Imputation (MI), developed by Rubin, has become a popular method for addressing the presence of missing data [1]. Multiple imputation entails the creation of M complete datasets, in which missing values have been replaced by plausible values generated from an imputation model.

An issue that has received relatively little attention in the methodologic literature is the effect of the prevalence of missing data on the quality of inferences made in the imputed datasets. In particular, little research has been conducted on the performance of MI when there is a high rate of missing data.

## Methods

We conducted an extensive series of Monte Carlo simulations to examine the effect of the percentage of subjects with missing data on one independent or explanatory variable on inferences about regression coefficients in a multivariable logistic regression model. The design of the simulations was informed by empirical analyses of patients hospitalized with acute myocardial infarction (AMI).

### Data for empirical analyses to inform the Monte Carlo simulations

We used data from the Enhanced Feedback for Effective Cardiac Treatment (EFFECT) Study [2], which collected data on patients hospitalized with AMI in Ontario, Canada between April 1, 1999 and March 31, 2001 and between April, 2004 and March 31, 2005. For the current study, data were available on 19,395 patients hospitalized with a diagnosis of AMI.

The outcome was a binary variable denoting death within one year of hospital admission. Outcome ascertainment was through linkage with the provincial death registry. Of the 19,395 patients, 3,898 (20.1%) died within one year of hospital admission.

We considered 10 variables for predicting 1-year mortality: age, systolic blood pressure at admission, heart rate at admission, hemoglobin (first recorded laboratory value after hospitalization), cholesterol (first recorded laboratory value after hospitalization), sex, angina, diabetes, history of previous AMI, and current smoker. The first five are continuous, while the last five are binary.

The outcome (one-year mortality), age, and sex were not subject to missing data (as they can be ascertained through linkages to provincial health insurance registries). Overall, 48.1% of patients were subject to missing data. The percentage of subjects who had missing data for the eight variables that were subject to missing data were: 0.9% (systolic blood pressure), 1.2% (heart rate), 1.2% (hemoglobin), 40.5% (cholesterol), 1.4% (angina), 0.4% (diabetes), 1.6% (previous AMI), and 13.4% (current smoker).

### Multiple imputation in empirical data

We used the multivariate imputation using chained equations (MICE) algorithm to create 48 complete datasets [3–5]. Predictive mean matching (PMM) was used for imputing missing continuous variables, while logistic regression was used for imputing missing binary variables. Based on a rule-of-thumb that the number of complete datasets should equal the percentage of subjects with any missing data, 48 complete datasets were created [6]. The imputation model for each variable that was subject to missingness used as predictors the other nine baseline covariates and the outcome for the final analysis model (1-year mortality). For example, the imputation model for current smoker used the other nine baseline covariates and 1-year mortality.

### Statistical analyses in the imputed empirical data to inform the design of the simulations

We conducted four sets of analyses in the complete datasets: (i) estimating the coefficients of the analysis model in which the odds of 1-year mortality was regressed on the 10 baseline covariates; (ii) estimating missing data models in which the odds of a variable being missing were regressed on the nine other baseline covariates and 1-year mortality; (iii) estimating the variance-covariance matrix of the ten baseline covariates; (iv) estimating the means and prevalence of each of the ten baseline variables.

In each of the 48 complete datasets, we regressed the binary outcome (1-year mortality) on the 10 baseline covariates described above. For simplicity, we assumed a linear relationship between each of the five continuous variables and the log-odds of mortality. The estimated regression coefficients and their standard errors were pooled across the 48 fitted models using Rubin’s Rules [1]. The resultant model will be used to generate outcomes in the simulations described below.

In each complete dataset, for each of the eight variables that had been subject to missingness in the original sample, we created a binary missing data indicator denoting whether that variable had been missing for the corresponding record in the original sample. We then estimated eight missing data models in each of the 48 complete datasets. Using logistic regression, the given missing data indicator was regressed on the other nine baseline covariates and the binary outcome variable from our analysis model (1-year mortality). Thus, in the case of systolic blood pressure, the binary indicator variable denoting whether systolic blood pressure was missing in the original sample was regressed on: age, heart rate at admission, hemoglobin, cholesterol, sex, angina, diabetes, history of previous AMI, current smoker, and 1-year mortality. Note that, in the case of systolic blood pressure, systolic blood pressure was not a covariate or predictor variable in the missing data model for systolic blood pressure. For a given variable, the coefficients of the missing data model were pooled across the 48 complete datasets using Rubin’s Rules. In the simulations described below, we will induce missingness in two variables using the cholesterol and smoking status missing data models. The c-statistics for these two models (pooled across the 48 complete datasets) were 0.67 and 0.71, respectively. The generalized R^2^ index for these two models were 0.12 and 0.11, respectively.

In each of the 48 complete datasets we computed the variance-covariance matrix of the 10 baseline covariates. These were then averaged across the 48 complete datasets. Finally, in each of the 48 complete datasets we determined the mean (for continuous variables) or prevalence (for binary variables) of the baseline covariates. These estimated means and prevalences were then averaged across the 48 complete datasets. The pooled prevalence of female sex, angina, diabetes, previous AMI, and current smoker were 36%, 32%, 27%, 24%, and 33%, respectively, across the 48 imputed datasets.

### Monte Carlo simulations: simulating a super-population

We designed a series of Monte Carlo simulations in which we examined the effect of the prevalence of missing data for one variable, with all other variables not being subject to missingness. This was done twice: first allowing only a continuous variable (cholesterol) to be subject to missingness; second allowing only a binary variable (current smoker) to be subject to missingness. The simulated super-population was designed to resemble the empirical data described in Data for empirical analyses to inform the Monte Carlo simulations and Multiple imputation in empirical data section. We simulate 10 baseline covariates and one binary outcome. For ease of description, we refer to each of the 10 simulated baseline covariates using the name of the variable in the empirical data that that simulated variable was intended to mimic.

For each subject in a super-population of size 1,000,000 we simulated 10 baseline covariates from a multivariate normal distribution with mean vector and variance-covariance matrix equal to those estimated in the previous section. The first five variables were retained as continuous while the last five variables were dichotomized. These last five variables were dichotomized using a threshold selected so that the prevalence of the resultant binary variable was equal to the prevalence of the corresponding binary variable estimated above. Thus, in the super-population, the 10 simulated baseline covariates had a multivariate distribution that was similar to that observed in the EFFECT AMI sample.

We then generated a binary outcome for each subject in the super-population. To do so, we applied the outcomes model (whose coefficients had been estimated in the empirical data above) to each subject in the super-population and computed the probability of the occurrence of the binary outcome. We then simulated a binary outcome from a Bernoulli distribution with this subject-specific parameter. We then fit the outcomes model in the simulated super-population by regressing the simulated binary outcome on the 10 simulated baseline covariates. The estimated regression coefficients will be considered the ‘true’ values of the regression coefficients to which the coefficients estimated below will be compared.

We then set the value of the cholesterol variable to missing for some subjects in the super-population. To do so, we used an iterative procedure to modify the intercept of the missing-data model for cholesterol, so that when the modified model was applied to the super-population, the prevalence of subjects with missing data on cholesterol was equal to the desired prevalence (${{\text{p}}_{{\text{missing}}}}$). The prevalence of missing cholesterol data in the super-population was determined as follows: for a given modified missing-data model, we applied the modified missing-data model for cholesterol to each subject in the super-population. We determined each subject’s probability of having missing cholesterol based on the applied model and generated a missing cholesterol indicator variable from a Bernoulli distribution with this subject-specific parameter. We then determined the proportion of subjects in the super-population for whom cholesterol was missing. Once the intercept of the missing data model had been determined, subjects for whom the missing data indicator was equal to 1 had their cholesterol value set to missing. The nine other baseline covariates were not subject to missingness. Using this approach, the relationship between the probability of cholesterol being missing and the nine other baseline covariates and the binary outcome reflected the relationships observed in the EFFECT AMI sample. The only difference was that the prevalence of missing data was set to a specified fixed prevalence. The missing data model for cholesterol contained the other nine baseline covariates and the binary outcome variable. Thus, the missing data mechanism for cholesterol was missing at random (MAR), as the probability of cholesterol was related to the other variables, but not to cholesterol itself.

The rationale for simulating a super-population from which subjects would be sampled was that this super-population could be constructed such that the prevalence of missing data was equal to a specified value.

### Monte Carlo simulations: statistical analyses

We drew a random sample of size N_sample_ without replacement from the super-population. Sampling without replacement was used to mimic what would be done in applied research, where random samples are typically drawn without replacement from the target population. As only one variable was subject to missingness, we used univariate imputation to impute the missing values of cholesterol in the random sample. The imputation model for cholesterol used the other 9 baseline covariates and the binary outcome. The number of complete datasets was set equal to the percentage of subjects with missing data [6] (to assess the validity of this approach when there was a high proportion of missing data, we examined one scenario with a sample size of 1,000, with 80% of the subjects were missing the continuous variable cholesterol and when parametric imputation was used. We then compared the use of M = 80, 160, and 240 imputed datasets. At most trivial differences in performance on all six performance metrics (see below for the six metrics) was observed). In each of the complete datasets we fit the analysis model in which a logistic regression model was used to regress the binary outcome variable on the 10 baseline covariates. The estimated regression coefficients and their standard errors were pooled across the complete datasets using Rubin’s Rules. Ninety-five percent confidence intervals were computed for each of the 10 estimated regression coefficients using Barnard and Rubin's small-sample degrees of freedom [7]. This process was repeated 1,000 times.

 The following analyses were then conducted: (i) computed the mean regression coefficient for each of the 10 baseline covariates across the 1,000 simulation replicates; (ii) determined the relative bias of the estimated regression coefficient for each of the 10 baseline covariates (by comparing the mean estimated regression coefficient across the 1,000 simulation replicates to the corresponding regression parameter that was used in the data-generating process); (iii) computed the standard deviation of the estimated regression coefficients for a given covariate across the 1,000 simulation replicates (this provides as an estimate of the sampling variability of the estimated regression coefficient); (iv) determined the average estimated standard error of each regression coefficient across the 1,000 simulation replicates; (v) compared the ratio of the quantity computed in (iv) to that computed in (iii) - this ratio should be equal to one if the standard errors are being estimated accurately; (vi) computed the mean squared error (MSE) of the estimated coefficients for each of the 10 covariates across the 1,000 simulations replicates. (vi) computed the empirical coverage rate of estimated 95% confidence intervals by determining the proportion of estimated confidence intervals that contained the true value that was used in the data-generating process.

### Factors in the Monte Carlo simulations

We allowed two factors to vary in our simulations: N_sample_ (the size of the random sample drawn from the super-population) and p_missing_ (the prevalence of missing data). We allowed the former to take five values: 500, 1,000, 5,000, 10,000, and 25,000. When N_sample_ was equal to either 500, 1,000, 5,000 or 10,000, we allowed p_missing_ to take values from 0.05 to 0.95 in increments of 0.05. When N_sample_ was equal to 25,000, we allowed p_missing_ to take values from 0.05 to 0.95 in increments of 0.10 (fewer values of p_missing_ were used due to the increased computational burden of the simulations in large samples).

When imputing missing values of cholesterol, parametric imputation was used in all of the above scenarios. We then repeated all scenarios using PMM for imputing missing values of cholesterol. When using PMM, Type 1 matching was used [8] and the size of the pool of potential matches was fixed at 5.

In all of the above simulations, only one continuous variable (cholesterol) was subject to missingness. None of other four continuous covariates, the five binary covariate or the binary outcome were subject to missingness. We then repeated all of the above simulations setting a binary variable (current smoker) to missing and having no missing data for any of the five continuous variables, the other four binary variables, or the binary outcome. Logistic regression was used for imputing missing values of smoking status. Apart from this modification, the simulations with missing smoking status was identical to those with missing cholesterol.

The simulations were conducted using the R statistical programming language (version 3.6.3). Univariate imputation was implemented using the mice function from the mice package (version 3.13.0).

## Results of Monte Carlo simulations

### A continuous covariate was subject to missing data

**Fig. 1:**
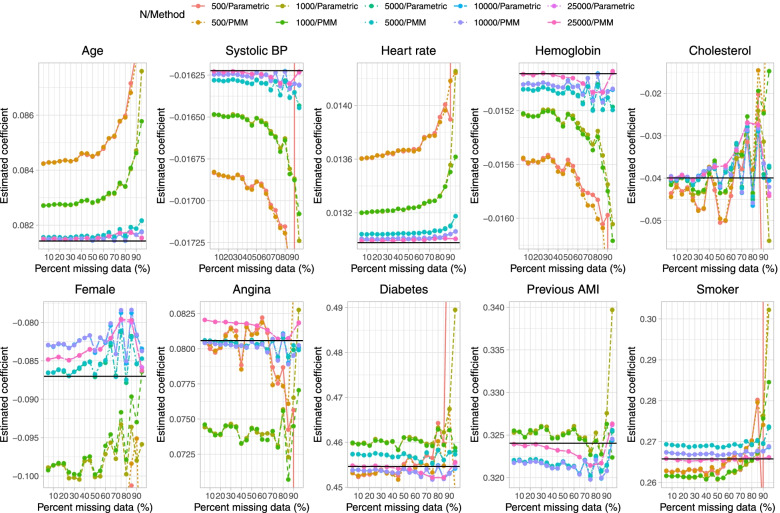
Mean estimated regression coefficient (when cholesterol is subject to missingness)

**Fig. 2:**
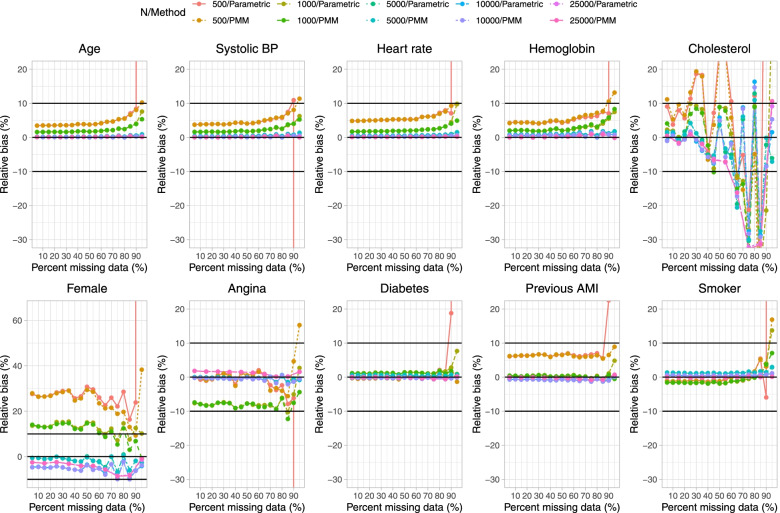
Relative bias in estimated regression coefficients (%) (when cholesterol is subject to missingness)

**Fig. 3:**
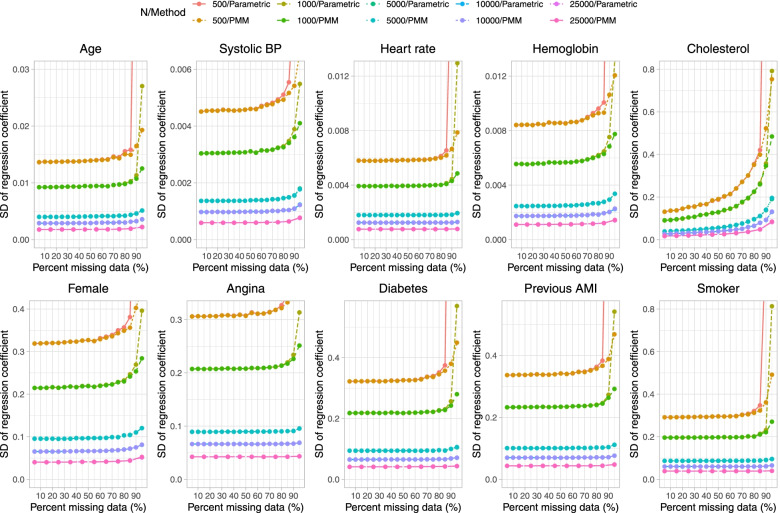
Standard deviation of estimated coefficient across simulation replicates (when cholesterol is subject to missingness)

**Fig. 4:**
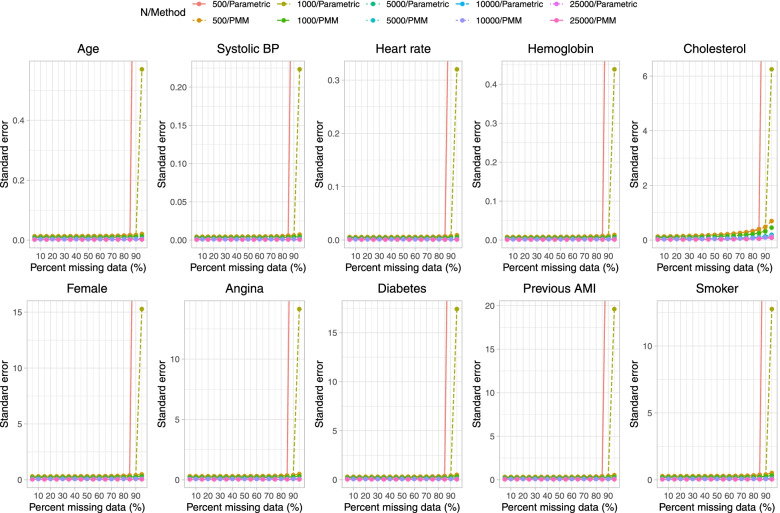
Estimated standard error (when cholesterol is subject to missingness)

**Fig. 5:**
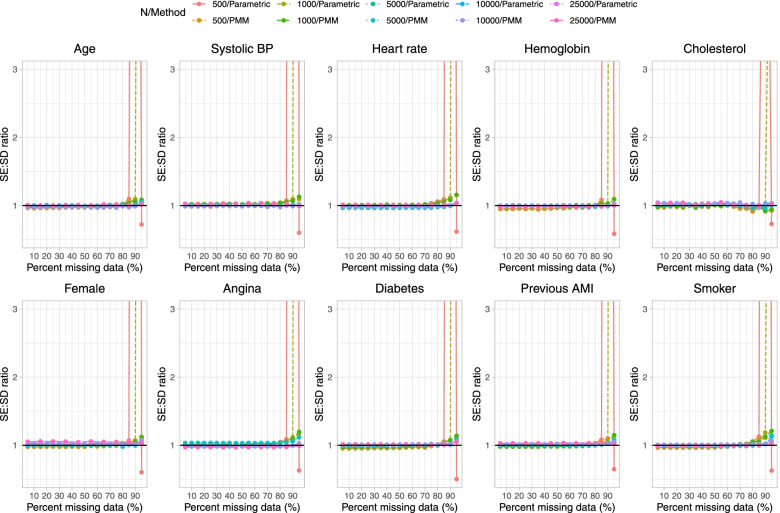
Ratio of estimated to empirical SE (when cholesterol is subject to missingness)

**Fig. 6:**
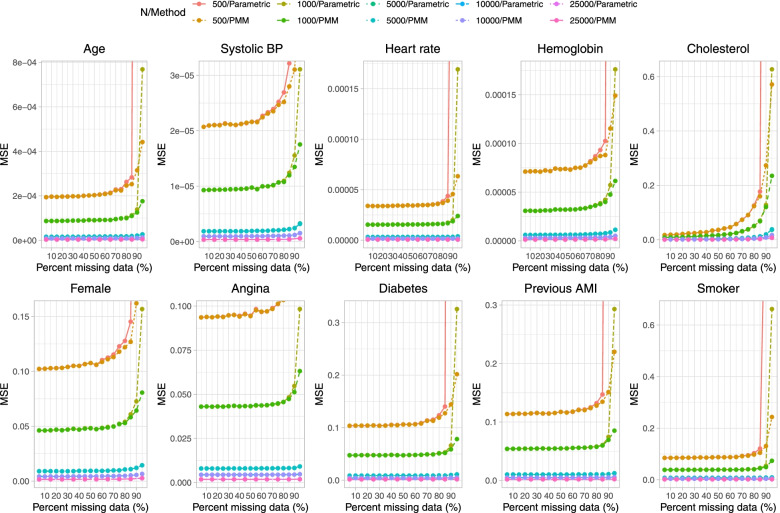
Mean squared error (when cholesterol is subject to missingness)

**Fig. 7:**
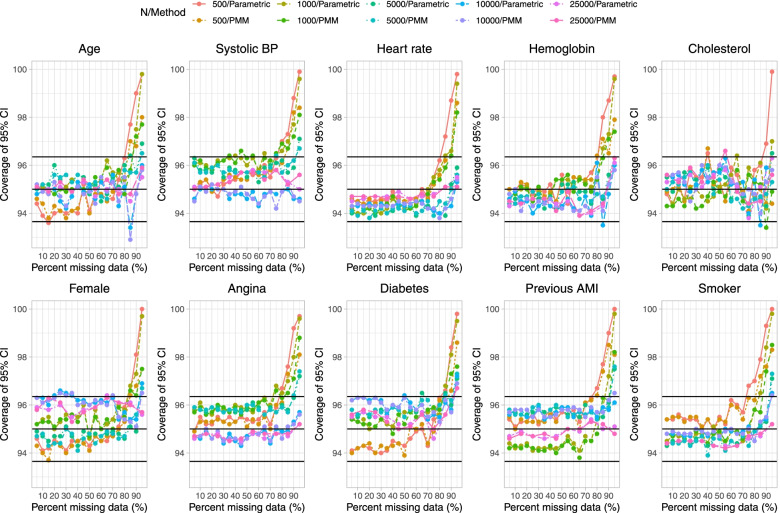
Empirical coverage rates of 95% CIs (when cholesterol is subject to missingness)

Results on estimation of the logistic regression model when a single continuous covariate (cholesterol) was subject to missingness are reported in Fig. 1 (mean estimated regression coefficient), Fig. 2 (relative bias), Fig. 3 (standard deviation of estimated regression coefficients across the 1,000 simulation replicates), Fig. 4 (mean estimated standard error of the estimated regression coefficients across the 1,000 simulation replicates), Fig. 5 (ratio of estimated to empirical standard error), Fig. 6 (MSE), and Fig. 7 (coverage of empirical 95% confidence intervals). We report on estimation of all 10 regression coefficients in the analysis model, not just the covariate that was subject to missingness. Each figure consists of 10 panels, one for each of the covariates in the analysis model. Note that in many panels we have truncated the vertical axes in order to improve legibility and the ability to discern trends.

On Fig. 1 we have superimposed a horizontal line on each panel denoting the true regression parameter when the regression model was fit to the super-population with no missing data, while on Fig. 2 we have superimposed three horizontal lines denoting relative biases of -10%, 0%, and 10%. In general, for those variables not subject to missingness (i.e., for all nine covariates apart from cholesterol), bias in the estimated regression coefficient tended to be minimal. The exception to this observation was when parametric imputation was used and the sample size was 500 and 95% of subjects were missing data on cholesterol. In general, across these nine covariates, bias tended to increase when the sample size was 500 or 1,000 and the prevalence of missing data was very high (≥ 90%). The regression coefficient for the variable subject to missingness (cholesterol) tended to be subject to moderate to large bias when the sample size was 500 or 1,000 and the prevalence of missing data was at least 85%. Moderate bias was observed even with very large sample sizes. For instance, when the sample size was 25,000 and the prevalence of missing data was 85%, the use of PMM resulted in a relative bias of -31.4% when estimating the regression coefficient for cholesterol.

In Fig. 3, we observe, as anticipated, that the standard deviation of the estimated regression coefficient across simulation replicates decreased with increasing sample size. When the sample size was 500 or 1,000, the standard deviation of the estimated regression coefficient tended to be substantially larger when parametric imputation was used and the prevalence of missing data was 95% compared to when the prevalence of missing data was less than 95%. This was true for all regression coefficients, not just for the regression coefficient for cholesterol. These results are mirrored by those observed in Fig. 4, in which the results for the mean estimated standard error are displayed. The estimated standard error increased exponentially when the sample size was 500 or 1,000 and parametric imputation was used when the prevalence of missing data was 90% or greater (when sample size was equal to 500) and when the prevalence of missing data was 95% (when sample size was equal to 1,000).

On Fig. 5 we have superimposed a horizontal line denoting a ratio of 1, indicating that the estimated standard error is accurately estimating the standard deviation of the sampling distribution of the regression coefficient. Our results suggest that estimation of the standard error is poor when the sample size was 500 or 1,000 and parametric imputation was used when the prevalence of missing data was 95%. Apart from this one scenario, our results suggest that estimation of standard errors tended to be relatively accurate even when the prevalence of missing data was very high.

The results for MSE (Fig. 6) suggest that MSE increased rapidly when the sample size was 500 or 1,000 and the prevalence of missing data was at least 90%. With all other sample sizes, MSE increased very gradually with increasing prevalence of missing data.

On Fig. 7 we have superimposed three horizontal lines denoting empirical coverage rates of 93.65%, 95%, and 96.35%. Given our use of 1,000 simulation replicates, empirical coverage rates that are less than 93.65% or greater than 96.35% are statistically significantly different from the advertised rate of 95% using a standard normal-theory test. In general, empirical coverage rates did not differ from their advertised rate except when the prevalence of missing data was very high. In these settings, coverage rates tended to be conservative, with the empirical coverage rate exceeding 95%. The degree of conservatism was greater when sample sizes were small.

### A binary covariate was subject to missing data

**Fig. 8:**
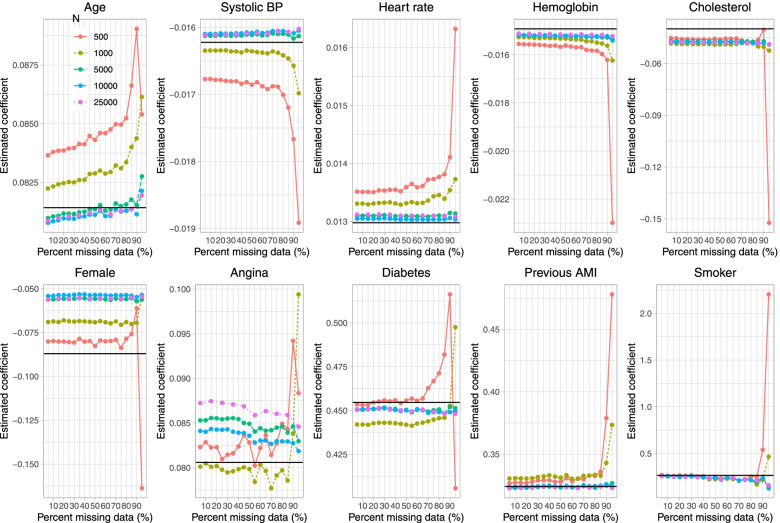
Mean estimated regression coefficient (when smoking is subject to missingness)

**Fig. 9:**
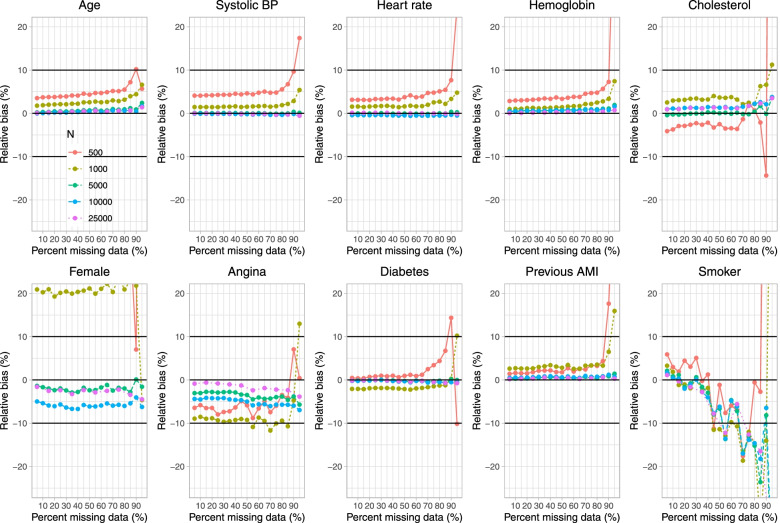
Relative bias in estimated regression coefficients (%) (when smoking is subject to missingness)

**Fig. 10:**
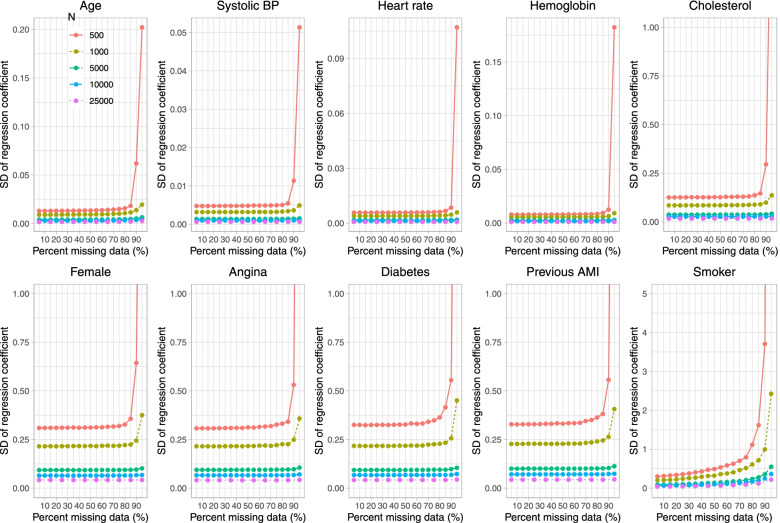
Standard deviation of estimated coefficient across simulation replicates (when smoking is subject to missingness)

**Fig. 11:**
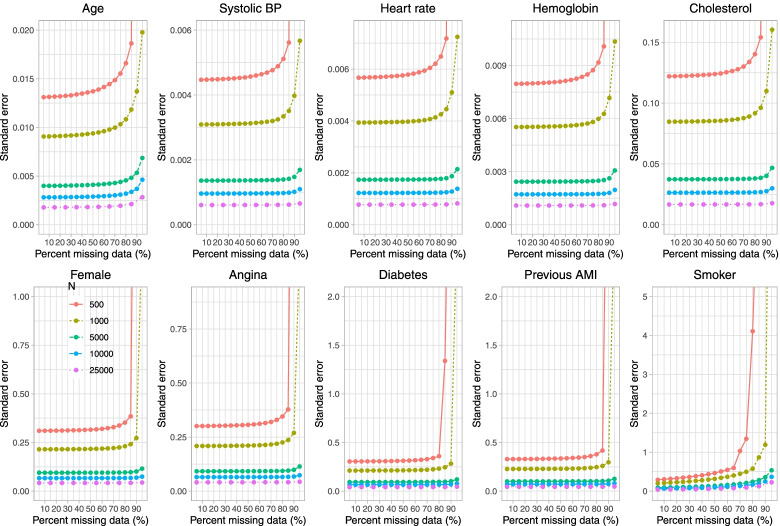
Estimated standard error (when smoking is subject to missingness)

**Fig. 12:**
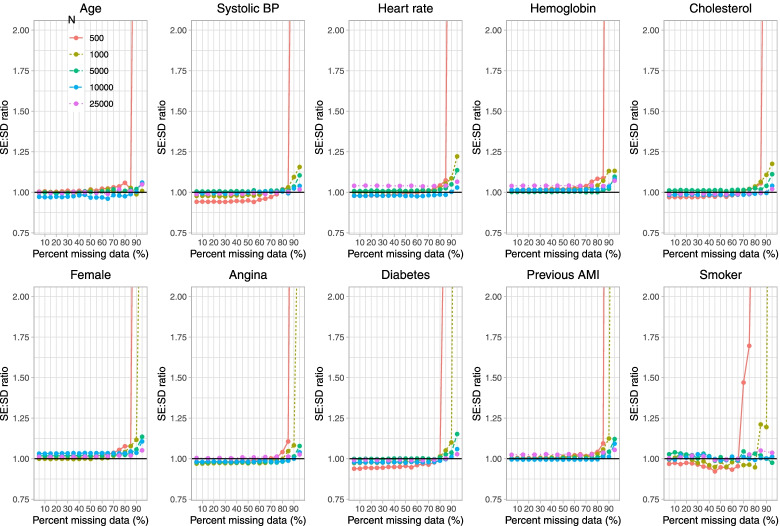
Ratio of estimated to empirical SE (when smoking is subject to missingness)

**Fig. 13:**
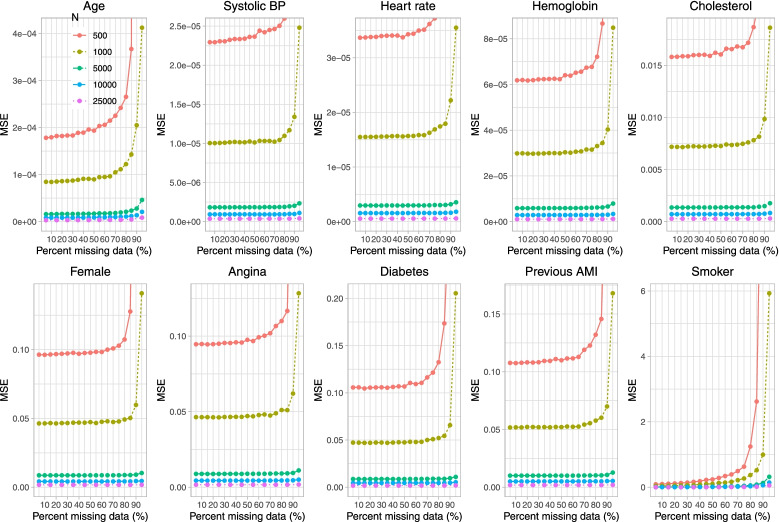
Mean squared error (when smoking is subject to missingness)

**Fig. 14:**
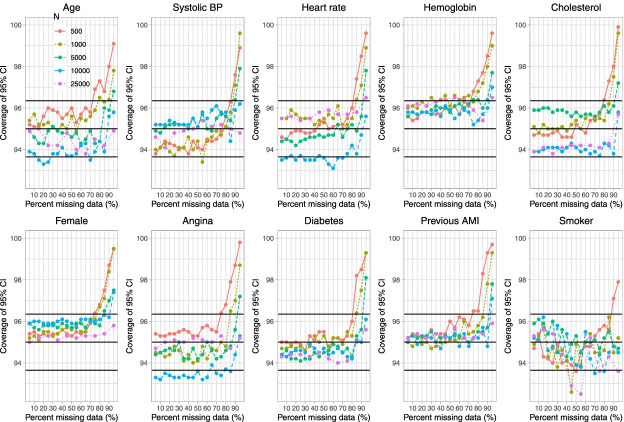
Empirical coverage rates of 95% CIs (when smoking is subject to missingness)

Results on estimation of the logistic regression model when a single binary covariate (current smoker) was subject to missingness are reported in Figs. 8, 9, 10, 11, 12, 13 and 14. These figures have a similar structure to those described above. Note that when imputing a missing binary variable, only parametric imputation was used and PMM was not used. In general, findings mirrored those observed in the preceding section when a continuous covariate was subject to missingness.

## Discussion

We examined the effect of the prevalence of missing data when estimating multivariable logistic regression models in samples created using multiple imputation. In general, we observed minimal bias in estimated regression coefficients regardless of the prevalence of missing data. When the sample size was small (1,000), the relative bias tended to increase when the prevalence of missing data was very high (≥ 90%). The standard deviation of the estimated regression coefficients across simulation replicates and the mean estimated standard errors tended to be very large when parametric imputation was used and the sample size was small (≤ 1,000) and the prevalence of missing data was 90% or greater. The standard errors were systematically mis-estimated in this specific scenario. In all other scenarios, standard errors were accurately estimated and were representative of the standard deviation of the sampling distribution of the regression coefficients. Finally, the empirical coverage rates of confidence intervals were close to the advertised levels, except when the prevalence of missing data was very high, in which case the estimated confidence intervals were conservative.

The mis-estimation of standard errors in the 95% missing scenario might not be the fault of multiple imputation and could well result from limitations of the complete-data analysis. For example, with *n* = 1,000 and with 95% of subjects missing data on smoking status, we have *n* = 50 subjects with observed data on smoking status. If 20% of the population smokes, then we expect *n* = 10 subjects who are current smokers. In that case, in about half of the 1,000 replications the number of observed smokers would be less than 10. A rule of thumb was proposed by Peduzzi and colleagues, who suggested that at least 10 outcome events were required for accurate estimation of the coefficients and standard errors in a logistic regression model [9]. In the setting described above, with, on average ten outcome events (current smoker) in the imputation model for smoking status, there may be an insufficient number of outcome events (current smoker) to result in accurate estimation of the regression coefficients and their associated standard errors. One needs only a handful of erratic estimates across the M imputed datasets to affect the overall estimate.

There is a limited literature on the use of MI in the presence of a high proportion of missing data. In a Master’s thesis, Lee examined the performance of MI when estimating a single mean [10]. The author considered a setting with sample sizes of 3,000 subjects and seven continuous variables that followed a multivariate normal distribution with mean zero and a specified variance-covariance matrix. The first variable was subject to missingness while the other six variables were not subject to missing data. The focus was on estimating the mean of the continuous variable that was subject to missingness. The percentage of missing data for this single variable ranged from 10 to 80% in increments of 10%. The author found the MI performed well, even when the prevalence of missing data was high, when the data were missing under a missing completely at random (MCAR) mechanism or a missing at random (MAR) mechanism. In a recent study, Madley-Dowd and colleagues examined the use of MI in the presence of a high prevalence of missing data [11]. They examined estimation of an analysis model using samples of size 1,000 in which the outcome variable was subject to missingness under either a MCAR or MAR mechanism; however, neither the single predictor variable nor the auxiliary variables were subject to missingness. The analysis model was a univariate linear regression model. The prevalence of missing data for the continuous outcome took on the following values: 1%, 5%, 10%, 20%, 40%, 60%, 80%, and 90%. They found that MI performed well at all levels of missingness. To the best of our knowledge, these are the only two studies that address the use of MI in settings which a high rate of missing data. Limitations of the first study include its focus on the estimation of a mean and that there was no examination of estimation of an analysis or outcome model. In many medical and epidemiological applications there is an outcomes model that is the primary focus of the research question. While the second study focused on an analysis model, the model was a univariate linear model and only the outcome variable was subject to missingness. In clinical research, the predictors are often subject to missingness. Thus, our current study fills a gap in the literature by considering a complex multivariable analysis model in settings in which one predictor variable was subject to missing data.

The current study is subject to certain limitations. First, like the two other studies described in the previous paragraph, our study relied on Monte Carlo simulations, and are thus dependent on the data-generating process that was used. However, a strength of our simulations was the use of a data-generating process that was based on empirical analyses of patients hospitalized with heart disease. Furthermore, we included both binary and continuous covariates, as these occur frequently in medical and epidemiological applications. A second limitation was that in all our simulations we fit a correctly specified imputation model. We thought that it was important to do so, as our intent was to examine the performance of MI in settings with a high prevalence of missing data. To do so, it is important to consider the ideal setting where everything is done correctly, and the only factor that varies is the proportion of missing data. In subsequent research, it would be important to consider the impact of using a mis-specified imputation model. We hypothesize that the effect of the rate of missing data will be amplified when a mis-specified imputation model is used. In other words, MI will perform relatively well when there is little missing data and a mis-specified imputation model is used, whereas it will perform poorly when there is a high rate of missingness and a mis-specified imputation model is used.

## Conclusions

Multiple imputation can be used to estimate the coefficients of a logistic regression model except when the sample is small and the prevalence of missing data is very high.

## Data Availability

The dataset from this study is held securely in coded form at ICES. While legal data sharing agreements between ICES and data providers (e.g., healthcare organizations and government) prohibit ICES from making the dataset publicly available, access may be granted to those who meet pre-specified criteria for confidential access, available at www.ices.on.ca/DAS (email: das@ices.on.ca).

## Abbreviations

AMI: Acute myocardial infarction
EFFECT: Enhanced Feedback for Effective Cardiac Treatment
MAR: Missing at random
MCAR: Missing completely at random
MI: Multiple imputation
MICE: Multivariate imputation using chained equations
MSE: Mean squared error
PMM: Predictive mean matching
